# Genome-wide variant-based study of genetic effects with the largest neuroanatomic coverage

**DOI:** 10.1186/s12859-021-04145-0

**Published:** 2021-04-30

**Authors:** Jin Li, Wenjie Liu, Huang Li, Feng Chen, Haoran Luo, Peihua Bao, Yanzhao Li, Hailong Jiang, Yue Gao, Hong Liang, Shiaofen Fang

**Affiliations:** 1 College of Automation, Harbin Engineering University, NO. 145 Nantong Street, Nangang District, Harbin, 150001 China; 2 Computer and Information Science, IUPUI, 723 W Michigan St, Indianapolis, IN 46202 USA

**Keywords:** Image genetics, Brain, Voxel, SNP, GWAS, Genetic algorithm

## Abstract

**Background:**

Brain image genetics provides enormous opportunities for examining the effects of genetic variations on the brain. Many studies have shown that the structure, function, and abnormality (e.g., those related to Alzheimer’s disease) of the brain are heritable. However, which genetic variations contribute to these phenotypic changes is not completely clear. Advances in neuroimaging and genetics have led us to obtain detailed brain anatomy and genome-wide information. These data offer us new opportunities to identify genetic variations such as single nucleotide polymorphisms (SNPs) that affect brain structure. In this paper, we perform a genome-wide variant-based study, and aim to identify top SNPs or SNP sets which have genetic effects with the largest neuroanotomic coverage at both voxel and region-of-interest (ROI) levels. Based on the voxelwise genome-wide association study (GWAS) results, we used the exhaustive search to find the top SNPs or SNP sets that have the largest voxel-based or ROI-based neuroanatomic coverage. For SNP sets with >2 SNPs, we proposed an efficient genetic algorithm to identify top SNP sets that can cover all ROIs or a specific ROI.

**Results:**

We identified an ensemble of top SNPs, SNP-pairs and SNP-sets, whose effects have the largest neuroanatomic coverage. Experimental results on real imaging genetics data show that the proposed genetic algorithm is superior to the exhaustive search in terms of computational time for identifying top SNP-sets.

**Conclusions:**

We proposed and applied an informatics strategy to identify top SNPs, SNP-pairs and SNP-sets that have genetic effects with the largest neuroanatomic coverage. The proposed genetic algorithm offers an efficient solution to accomplish the task, especially for identifying top SNP-sets.

**Supplementary Information:**

The online version contains supplementary material available at 10.1186/s12859-021-04145-0.

## Background

With recent technological advances in acquiring multimodal neuroimaging and high-throughput genetics data, massive amounts of multimodal structural and functional imaging data on the human brain as well as genome-wide genetic data from the same set of subjects have been collected. With the availability of these data sets, brain imaging genetics has becoming an emerging research area, which provides enormous opportunities for examining the effects of genetic variations on the brain. Many studies have shown that the structure, function and abnormality (e.g., those related to Alzheimer’s disease) of the brain are heritable. However, which genetic variations contribute to these phenotypic changes are not completely clear.

To bridge this gap, a number of approaches for finding associations between genetic variations and imaging phenotypes arise. A genome-wide association study (GWAS) [1] conducted by Christopher et al., which links genetic variations such as single nucleotide polymorphisms (SNPs) to imaging phenotypes, mainly analyzed the association between SNPs with measures at regions of interest (ROIs). The voxelwise GWAS (vGWAS) was proposed by Stein et al. [2, 3] to generate detailed three-dimensional maps of the SNP effects on the brain, without requiring defining ROIs on the brain. Huang et al. [4] developed a functional genome-wide association study (FGWAS) method to identify sparse signals in an extremely large search space. Compared to GWAS, FGWAS could improve detection capabilities to discover important genetic variations and gene-environment interactions that affect brain structure and function. Vounou et al. [5] proposed another method for simultaneously selecting SNP variants and binding regions assuming that the signals are sparse. This could reduce the number of SNPs and phenotypes tested. Among these methods, the voxelwise GWAS makes it possible to study the SNPs from a more nuanced perspective, and can capture subtle signals that are easily missed by ROI-based methods [6–8].

As the number of SNPs increases, the amount of data increases exponentially. In prior studies, the researchers used a variety of methods to detect two marker effects [9]. Günther F, et,al. built models using neural networks to reveal the effects. There is a problem that the estimated weight cannot be explained [10]. The random Forests was used to build accurate decision trees for the effects [11] and the two-stage grouped sure independence screening [12] was used to detect the causal interactions. To detect the effects, other methods had been devised, such as odds ratio [13], Ant Colony Optimization Algorithm [14] and MegaSNPHunter [15]. However, since the effects of n SNPs is more complicated and the data increases rapidly, the research on it is still a problem to be developed.

Although brain imaging genetics has become an emerging and rapidly growing research field [16–19], the study of genetic effects on neuroanatomic coverage remains to be an underexplored topic. To bridge the gap, in this paper, we perform a genome-wide variant-based study, and aim to identify top SNPs or SNP sets which have genetic effects with the largest neuroanotomic coverage at both voxel and ROI levels. Based on the voxelwise GWAS results, we use the exhaustive search to find the top SNPs or SNP sets that have the largest voxel-based or ROI-based neuroanatomic coverage. For SNP sets with >2 SNPs, we propose an efficient genetic algorithm to identify top SNP sets that can cover all ROIs or a specific ROI.

## Results

### Results of single marker effects

**Fig. 1:**
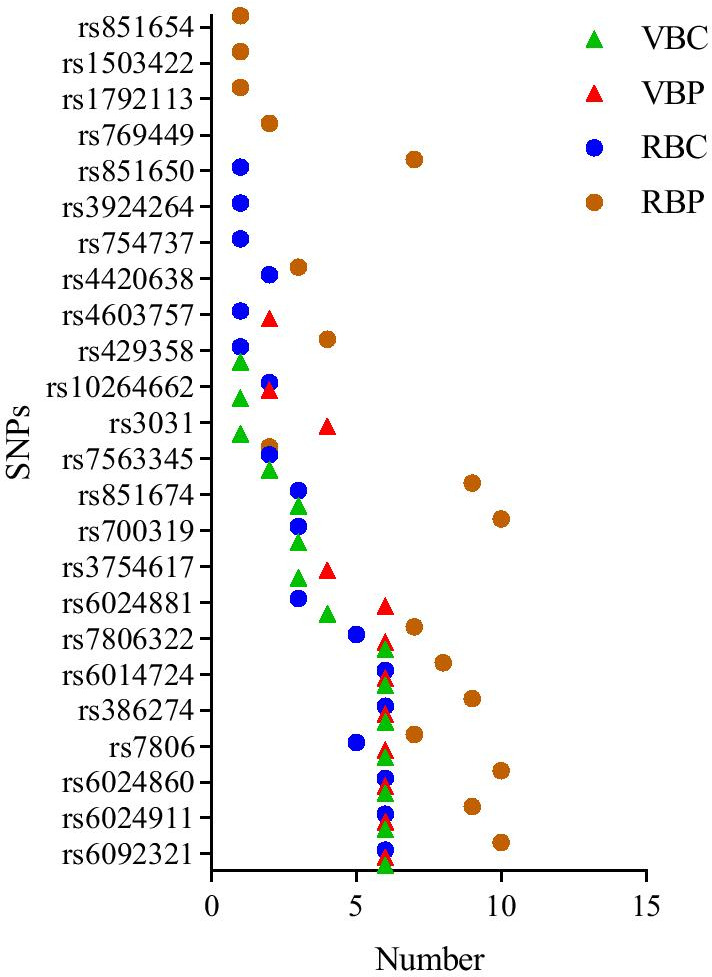
The top 10 SNPs and their frequency in VBC, VBP, RBC, RBP. The X axis represents the number of top 10 SNPs in all set thresholds; The Y axis represents the name of SNPs; VBC = Ranking SNPs according to the number of covered voxels; VBP = Ranking SNPs according to minimally required p threshold; RBC = Ranking SNPs according to the number of ROIs; RBP = Ranking SNPs according to minimally required p threshold

We presented the frequency of the top 10 SNPs with different thresholds from VBC, VBP, RBC and RBP in Fig. 1. Twenty-four SNPs exhibited large neuroanotomic coverage. As expected, the most frequent loci were identified on chromosome 20, including rs6092321 from the *RTF2* region, and rs6024860 (N/A). Other SNPs identified in this study are shown in Fig. 1. Table 1 shows the variances explained by identified SNPs. The main effects of rs6092321 and rs6024860 account for 0.93% and 0.82% of phenotypic variance respectively.

### Results of SNP–SNP effects

**Fig. 2:**
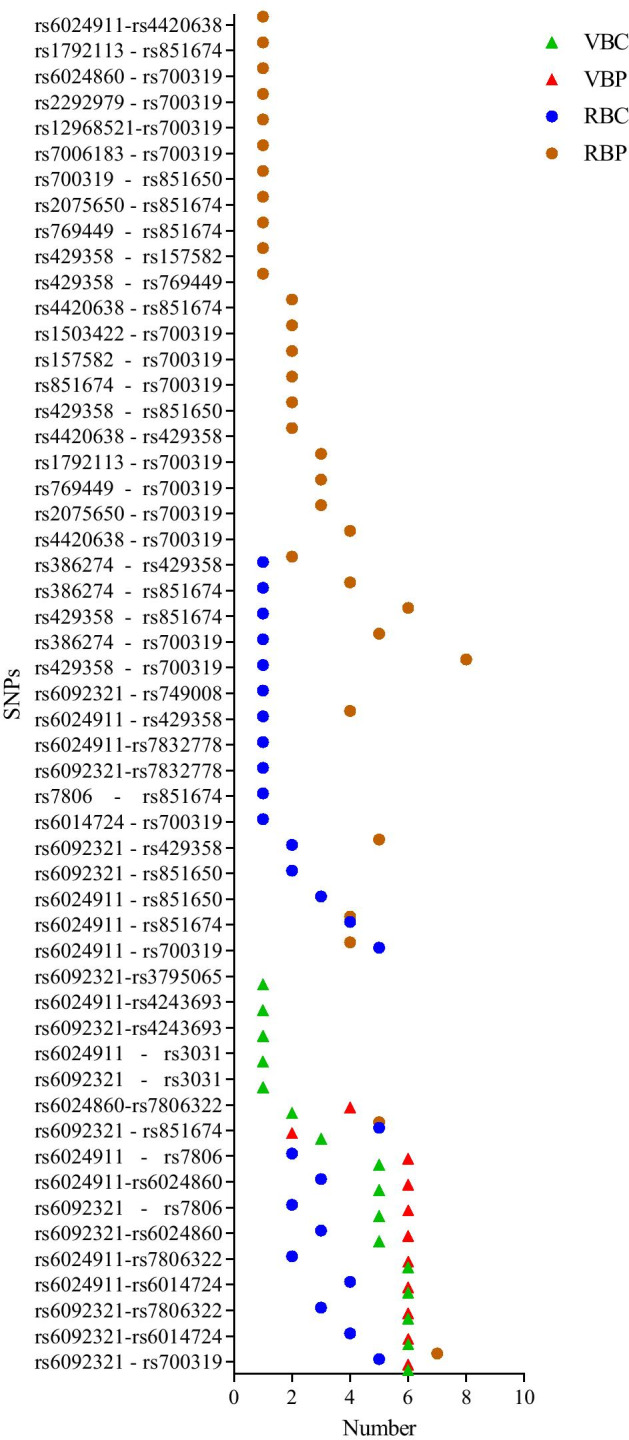
The top 10 SNP pairs and their frequency in VBC, VBP, RBC, RBP. The X axis represents the number of top 10 SNPs in all set thresholds; The Y axis represents the name of SNP pairs; VBC = Ranking SNPs according to the number of covered voxels; VBP = Ranking SNPs according to minimally required p threshold; RBC = Ranking SNPs according to the number of ROIs; RBP = Ranking SNPs according to minimally required p threshold

Fifty-three pairs of SNPs showed statistically significant effects on neuroanotomic coverage. Only 1 pair passed the covering criterion: all the four strategy are required to be covered by the SNP pair. The result of SNP–SNP effects was rs6092321 (*RTF2*) - rs700319 (*CNTNAP2*). Figure 2 provided the frequency of other SNP pairs. The variance explained by rs6092321 - rs700319 is 0.94%, and the correlation of rs6092321 - rs700319 are 0.0945, 0.0533 and $-0.0381$ (Table  1).

### Results of three SNPs effects

**Fig. 3:**
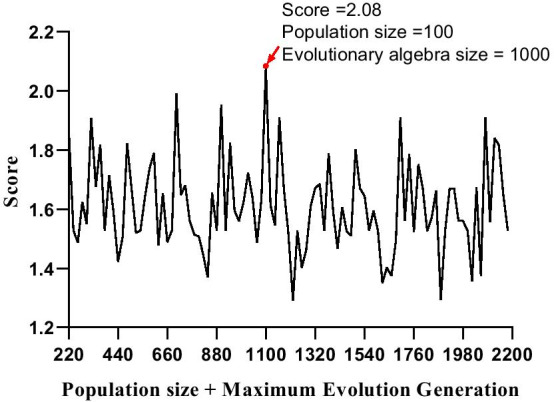
The relationship between score and Mixed parameters (population size + maximum evolution generation). The X axis represents the score of SNP sets; The Y axis represents the sum of population size and maximum evolution generation

To find a suitable population size and a maximum evolutionary generation, we chose 100 and 1000 as the center, 0–200 and 0–2000 as the range, and 20 and 200 as the step size to calculate the scores separately. To avoid the occasional final score being too small, we counted the maximum score and the corresponding SNP set after 10 cycles in each case. The resulting score, population size and maximum evolutionary generation are shown in Fig. 3. As shown in Fig. 3, when we used the 100 (population size) and 1000 (maximum evolutionary generation) to filter the SNP sets, the score of the SNP sets reached the highest value of 2.08.

**Fig. 4:**
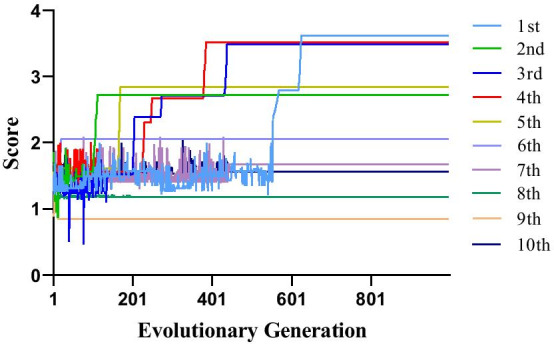
The curve of score and evolutionary generation. The population size is 100; The maximum evolutionary generation is 1000; 1st, 2nd, 3rd, 4th, 5th, 6th, 7th, 8th, 9th and 10th are the number of times in 10 cycles

With the selected population size (100) and the maximum evolutionary generation (1000), we counted the relationship between the score and evolutionary generation. The resulting curve is shown in Fig. 4. To avoid the occasional final score being too small or big, we ran the genetic algorithm for 10 times with the same parameters. The scores of the 6th, 7th, 8th, 9th and 10th are around 1.5, and the 6th and 9th converge prematurely. The defects in the initial population and in the offspring may be the major reason. All the remaining scores are above 2.0, and the 1st, 3rd and 4th have the better scores. Their initial scores fluctuate drastically, and the scores increases rapidly over time. Wherein the 1st at about 600th generation converges to optimal score, and the turning point of 3rd and 4th are around 400th generation. This is because the parent with better score that would have an opportunity to breed and pass on their codes.

**Fig. 5:**
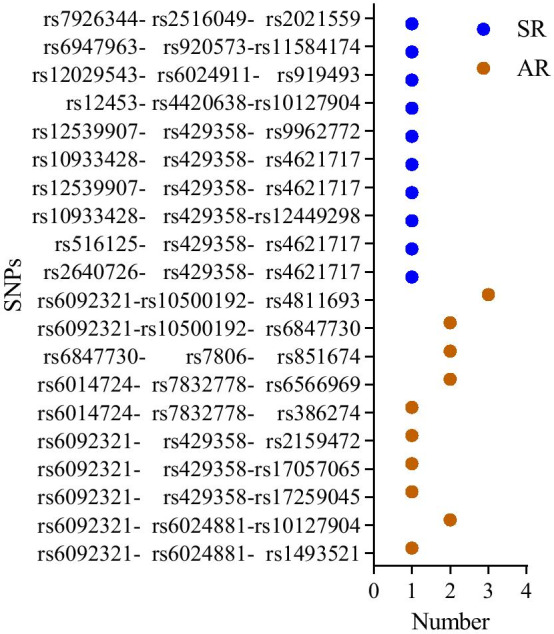
The top 10 SNP sets and their frequency in AR and SR. The X axis represents the number of top 10 SNPs in all set thresholds; The Y axis represents the name of SNP sets; AR = Ranking SNPS according to minimal p threshold covered all ROIS; SR = Ranking SNPS according to minimal p threshold covered a specific ROI

As expected, the SNP sets with largest neuroanotomic coverage, minimum *p* value and high number from AR included rs6092321 (Fig. 3). With regard to SR (two ROIs were hippo-campus_L and hippo-campus_R in our experiment), rs429358 was found in most identified SNP sets with the minimum *p* value (Fig. 5). The results of three SNPs effects were rs6092321 (*RTF2*) - rs10500192 (*CNTNAP2*) - rs4811693 (*FAM210B*) from AR, and rs429358 (*APOE*) - rs2640726 (*EPHX2*) - rs4621717 (*CNTNAP2*), rs429358 (*APOE*) - rs516125 (*SCARA3*) - rs4621717 (*CNTNAP2*), rs429358 (*APOE*) - rs10933428 (*INPP5D*) - rs4621717 (*CNTNAP2*) and rs429358 (*APOE*) - rs12539907 (*CNTNAP2*) - rs4621717 (*CNTNAP2*) from SR. Details are available in Table  1.

### Post hoc analysis

**Table 1:** Eight significant SNPs, SNP pair and SNP sets identified in VBC, VBP, RBC, RBP, AR and SR

NO.	SNP	Gene	CHR	Explained variance	Pearson correlation		
				(R square)	Hippocampus	Memories	Memory
1	rs6092321	*RTF2*	20	0.009303	0.073	0.0806	0.0042
2	rs6024860		20	0.008197	0.0198	0.075	0.0354
3	rs6092321-	*RTF2*	20	0.00939	0.0945	0.0533	− 0.0381
	rs700319	*CNTNAP2*	7				
4	rs6092321-	*RTF2*	7	0.009825	0.0804	0.0675	− 0.0111
	rs10500192-	*CNTNAP2*	7				
	rs4811693	*FAM210B*	20				
5	rs429358-	*APOE*	19	0.0112	0.3034	0.1542	0.1274
	rs2640726-	*EPHX2*	8				
	rs4621717	*CNTNAP2*	7				
6	rs429358-	*APOE*	19	0.0112	0.3094	0.1463	0.1198
	rs516125-	*SCARA3*	8				
	rs4621717	*CNTNAP2*	7				
7	rs429358-	*APOE*	19	0.0113	0.3098	0.147	0.116
	rs10933428-	*INPP5D*	2				
	rs4621717	*CNTNAP2*	7				
8	rs429358-	*APOE*	19	0.0117	0.3085	0.1522	0.1186
	rs12539907-	*CNTNAP2*	7				
	rs4621717	*CNTNAP2*	7				

Explained variance = For 1, 2, 3 and 4, explained variance of whole brain; for 5, 6, 7 and 8, explained variance of hippocampus; Pearson correlation = the association between SNPs, SNP pair or SNP sets and features; Hippocampus, Memories and Memory = the part of features associated with Alzheimer’s disease

Table  1 also shows the correlation between SNPs and hippocampus, memories and memory. For each SNP, SNP pair and SNP set, we superimposed the voxel images of each group of SNPs. We combined the images and features [20] to determine the contribution of SNPs to brain features. For rs6092321, the correlation account for 0.073, 0.0806 and 0.0042, the correlation of rs6092321 - rs700319 account for 0.0945, 0.0533 and $-0.0381$, and the correlation of rs6092321 - rs10500192 - rs4811693 are 0.0804, 0.0675 and -0.0111. In SR, the correlation of rs429358 - rs12539907 - rs4621717 are 0.3085, 0.1522 and 0.1186.

## Discussion

In this work, we performed voxelwise GWAS and using a sample of 1515 subjects from the Alzheimer’s Disease Neuroimaging Initiative (ADNI) database. To our knowledge, this study on detecting SNP, SNP pairs and SNP sets is the first study of genetic effects on neuroanatomic coverage.

The down-sampled image were obtained under the different treatments and used for vGWAS with the genetic data. Then the resulting data analyzed using four computational programs (VBC, VBP, RBC, and RBP) for ranking the SNPs. SNPs were selected based on the number of voxels less than the set *p* value threshold in VBC, and VBP served as a control group to filter SNPs based on the *p* value corresponding to the given number of voxels. The primary purpose of VBP is to find the “missing SNPs” in VBC. As illustrated in Fig. 1, the top 9 SNPs are the same and a few SNPs are different in VBC and VBP. The difference of *p* value in voxels of SNPs accounts for the major cause. Selecting SNPs that affect ROIs and differ with chosen SNPs on voxel level is the main objective in RBC and RBP. The ROI coverage was added to RBC as an additional condition on the basis of VBC. Similar to RBC, the given number of voxels was changed to the set number of ROIs, and the coverage of ROI was added in RBP. Picking SNPs from another condition and comparing them with SNPs in RBC are the primary aim of RBP. A similar discrepancy among SNPs was observed on voxel level and ROI level and directly correlated to the addition of ROI coverage.

According to the four different programs, different rankings of the top SNPs were shown and several missing SNPs were found. These highlight the necessity of utilizing multiple procedures to obtain the best possible SNPs. The frequency of the SNPs can be observed directly by the number, and the lower values indicate “missing SNPs” that have been recovered.

For the number of SNP set selected equal or greater than 3, the number of SNP sets reaches 900 million, and the time of exhaustive strategies increases exponentially. Therefore, algorithms to shorten the time or reduce the data set was developed. Genetic algorithm is suitable to resolve the issue. The initial population of genetic algorithm can be considered a reduced data set, and the offspring after cross-inheritance can be treated as a new data set that is constantly changing. Importantly, when an offspring with a large score appears, the data set will quickly converge to this score, which greatly shortens the selection time. As shown in Fig. 4, the dramatic shifts in scores are about 3.5 and 2.8, and the scores afterwards plateau at this value. And the time for 1000 evolutionary generation can be shortened to less than 20 min.

In single-marker analysis, the most obvious SNP identified from the analysis is rs6092321 (within the *RTF2* gene on chromosome 20). The ROIs affected by SNP rs6092321 (*RTF2*) with a high coverage are left gyrus rectus, right gyrus rectus, left entorhinal cortex, right entorhinal cortex, and vermis_9. The specific function for left gyrus rectus and right gyrus rectus has not yet been brought to light. However, subgroup analysis showed the negative impact of gyrus rectus resection on language and memory recall categories [21]. Ballmaier M. et al. had reported that very significant strong gray matter defects were observed in the gyrus rectus [22]. The volume of the left entorhinal cortex was different between progressive (Alzheimer’s disease) and stable mild cognitive patients [23]. MRIs show that Gomez-Lopez-Hernandez syndrome is characterized by cerebellar sacral loss or partial cerebellar loss and varying degrees of cerebellar fusion [1]. In terms of images, after calculating the Pearson correlation between rs6092321 (*RTF2*) and features (hippocampus, memories, memory and speaker), we found that rs6092321 (*RTF2*) is positively correlated with hippocampus, memories, memory and speaker (Additional file 1). This gives an affirmation of the impact of rs6092321 on ROIs above.

In SNP–SNP analysis, rs700319 (within the *CNTNAP2* gene on chromosome 7) and rs6092321 (*RTF2*) appear in pairs with highest frequency, since the frequency of 700319 (*CNTNAP2*) in single marker analysis was not high. The variances explained by rs6092321 (*RTF2*) - rs700319 (*CNTNAP2*) is bigger than rs6092321 alone. Combined with additional file 1, these give the evidence that rs700319 (*CNTNAP2*) is another important SNP. Comparing with rs6092321 (*RTF2*), rs6092321 (*RTF2*) - rs700319 (*CNTNAP2*) is positively correlated with memories and hippocampus, and negatively correlated with memory (Table  2).

Although the results obtained by the genetic algorithm are not the global optimal solutions, the running time is reduced greatly. And the result achieves improvement with the increase of running time. In AR, considering the variances explained by rs6092321 (*RTF2*) and rs6092321 (*RTF2*) - rs10500192 (*CNTNAP2*) - rs4811693 (*FAM210B*) and the additional file 1, rs10500192 (*CNTNAP2*) and rs4811693 (*FAM210B*) are the “missing SNPs” for the genetic effects on neuroanatomic coverage.

In SR, we found a sudden increase in the number of rs429358 (*APOE*). A meta-analysis estimated that the ratio of homozygous rs429358 (C;C) individuals to the more common ApoE3 / ApoE3 homozygote was 12 times of late-onset Alzheimer’s disease and 61 times of early-onset disease [24]. These results confirm our prediction and prove that the candidate SNPs we selected will provide more valuable information. For the SNP sets including rs429358 (*APOE*), the pearson correlation between SNP sets and features (hippocampus, memories and memory) show that the SNP sets have a positive correlation on these features. This suggests that SNP group makes sense for hippocampus, and memory and is also consistent with our initial vision. Considering the difference among the SNP sets including rs429358 (*APOE*) and the additional file 1, rs12539907 (*CNTNAP2*) is a “missing SNP” for hippocampus.

Based on the above, the loci including the “missing SNPs” identified in our analysis are *CNTNAP2* and *FAM210B*. *CNTNAP2* has an pivotal effect in maintaining normal network activity and synaptic transmission. The transsynaptic bridge formed by *CNTNAP2* on the presynaptic membrane and *CNTN2* on the post-synaptic membrane can spans the synaptic cleft [25–27]. The dendritic arborization and the numbers of excitatory synapses, inhibitory interneurons, and inhibitory synapses were all reduced by the loss of *CNTNAP2* [28–30]. *CNTNAP2* guides the cellular migration of neurons to their correct position in the brain [28, 31]. The impact of *CNTNAP2* on cellular migration of neurons, synapse development, and synaptic communication indicate that it plays a key role in the brain function. *FAM210B* can promote the transfer of protoporphyrinogen IX (PPIX) to FECH, and promote the introduction of iron and the synthesis of heme by forming oligomers with PPOX and FECH to enhance the introduction of mitochondrial iron and the synthesis of heme [32]. Stabilization of FECH protein caused by the binding of iron-sulfur clusters [33] or the increased transcription of FECH mRNA [34] lead to ferrochelatase protein expression increased during erythropoiesis. FAM210B can effectively transport iron to FECH, and / or affect the allosteric activation of the FECH enzyme [32]. The possible mechanisms behind *APOE*-*CNTNAP2* and *RTF2*-*CNTNAP2* warrant further investigation.

In summary, some of the SNPs and genes identified in our analysis have shown interesting associations with the genetic effects on neuroanatomic coverage from prior knowledge of current literatures, such as rs6092321, rs429358, *RTF2*, *APOE* and *CNTNAP2*. The additional file 1 showed the correlation between the brain structure of identified SNPs, SNP pair and SNP sets and the features provide by [20]. These results were very encouraging and confirmed that the analysis was successful as it was able to identify the “missing SNPs” and the top SNPs that have largest neuroanatomic coverage. In addition, numerous SNPs, SNP pairs and SNP sets revealed in our study had genetic effects, which warrant further investigation or replication in future studies.

The limitations of our study are as follows: (1) We examined 1784 SNPs. We also need use more SNPs to analyze. (2) We used the genetic algorithm to analyze the effects of multiple SNPs. The result is a local optimal solution and more effective and efficient strategies are still to be developed in multiple SNPs. (3) Comparing the exhaustive search, we can get better results in less time using genetic algorithms. However, the results get better with time, and users still have to wait a long time to get better results. (4) When a better set of SNPs appears, the offspring will be stuck in this combination.

## Conclusion

Aiming at studying the relationship between SNPs and brain structures, we performed voxel-wise GWAS and SNPs analysis to discover the SNPs which could affect more areas of the brain based on ROI and voxel using a sample of 1515 subjects from the ADNI database. The single-marker analysis identified the SNPs rs6092321 and rs6024860, which contributed the highest genetic effects on neuroanatomic coverage in all case. The SNP–SNP analysis identified new SNP pair including rs6092321 in single-marker analysis, which showed strong associations with the neuroanatomic coverage. This was rs6092321 and rs700319. The n SNPs analysis identified a number of novel findings, which showed higher associations with whole brain or hippocampus. Perhaps what is more important in this study is the discovery of SNPs that has not yet been associated with the Alzheimer’s Disease (AD) in conventional GWAS studies. Based on voxelwise GWAS, the effects of n SNPs and SNP–SNP showed high-level statistical significance than the single-marker effects. These may help address part of missing SNPs and brain clusters. Although this study focuses on SNPs effects, the findings may well show that the genetic algorithm is an interesting method for detecting the effects of n SNPs.

Our voxelwise genome-wide association study and genetic effects study on neuroanatomic coverage have the following strengths in addition to the above interesting findings. (1) To our knowledge this is the first study of genetic effects on neuroanatomic coverage. (2) Using voxelwise volumetric measurements as phenotypes confers higher statistical power than using conventional phenotypes and is able to find the “missing SNPs”. (3) The sample in this study included HC, SMCI, EMCI, LMCI, and AD, thus providing a rank-ordered spectrum of the disease progression. (4) Our approach is more computationally efficient than the exhaustive strategies, facilitating the analysis of genome-wide SNPs effects.

## Methods

We first describe the imaging and genotype data used in this work and then present our methods.

### Imaging and Genotype Data

The imaging and genotype data of 1,515 non-Hispanic Caucasian subjects were downloaded from http://adni.loni.usc.edu. In this work, we analyzed the MRI scans and genotyping data, including 353 healthy control (HC), 89 significant memory concern (SMC), 273 early MCI (EMCI), 504 late MCI (LMCI), and 296 AD participants. The characteristics of these 1,515 subjects are shown in Table 2.

**Table 2:** Participant characteristics

Subjects	HC	SMC	EMCI	LMCI	AD
Number	353	89	273	504	296
Gender (M/F)	187/166	36/53	153/120	309/195	166/130
Age(mean±sd)	$74.9\pm 5.7$	$72.2\pm 5.7$	$71.3\pm 7.1$	$74.0 \pm 7.6$	$74.7 \pm 7.6$
Edu(mean±sd)	$16.1\pm 2.7$	$16.8\pm 2.6$	$16.1\pm 2.6$	$16.0 \pm 2.9$	$15.5\pm 2.9$

HC = Healthy Control; SMC = Significant Memory Concern; EMCI = Early Mild Cognitive Complaint; LMCI = Late Mild Cognitive Complaint; AD = Alzheimer’s Disease

Preprocessed T1-weighted volumetric MRI scans were aligned to each participant’s same visit scan and normalized to the Montreal Neurological Institute (MNI) space. Voxel-based morphometry (VBM) was applied on MRI scans to extract voxel-wise volumetric measurements. Briefly, scans were aligned to a T1-weighted template image, segmented into gray matter, white matter and cerebrospinal fluid maps, and then normalized to the MNI space. The gray matter density (GMD) maps were extracted and smoothed with an 8mm FWHM kernel. The resulting GMD images are then down-sampled to a dimension of $61 \times 73 \times 61$ (i.e., containing 271,633 voxels) to reduce the computation cost in subsequent analyses. The 116 ROIs and their coordinates were anatomically defined using the Automatic Anatomical Labeling (AAL) atlas [35], and registered with the down-sampled images.

Genotyping data was processed as described in [36, 37], which resulted in 565,373 SNPs for all 1515 participants. A list of 24 AD candidate genes from a prior large scale GWAS meta-analysis [38] were analyzed in this study. We extracted SNPs located in $\pm 20$K bp of the 24 AD genes, and finally included total 1784 SNPs in our imaging genetic association analysis.

### Overall strategy

As shown in Fig. 4, the first step of our investigation is to perform pairwise univariate voxelwise genetic association analysis on 1515 subjects to examine the variant effect of 1784 SNPs on 271,633 voxels of the brain. The *p* value of each SNP-voxel pair was first obtained by performing genetic association of all the voxels for each studied SNP. Using these voxelwise SNP results, we implemented the following four strategies to identify top SNPs or SNP pairs that affect the largest portion of the neuroanatomy on the voxel or ROI basis.

1. *VBC*: Given a p threshold, rank SNPs according to the number of significant voxels.
2. *VBP*: Given a voxel number threshold, rank SNPs according to minimally required p threshold.
3. *RBC*: Given a p threshold and ROI coverage threshold, rank SNPs according to the number of ROIs covered by an enough number of significant voxels.
4. *RBP*: Given an ROI number threshold and ROI coverage threshold, rank SNPs according to minimally required p threshold.

Although the exhaustive search strategy can be applied to identify top SNPs or SNP pairs, it won’t work on identifying SNP sets containing three or more SNPs (denoted as high-order SNP sets for convenience) due to exponentially increasing computational cost. To address this challenge, we propose a more efficient genetic algorithm to identify top high-order SNP-sets, whose effects have the largest neuroanatomic coverage.

**Fig. 6:**
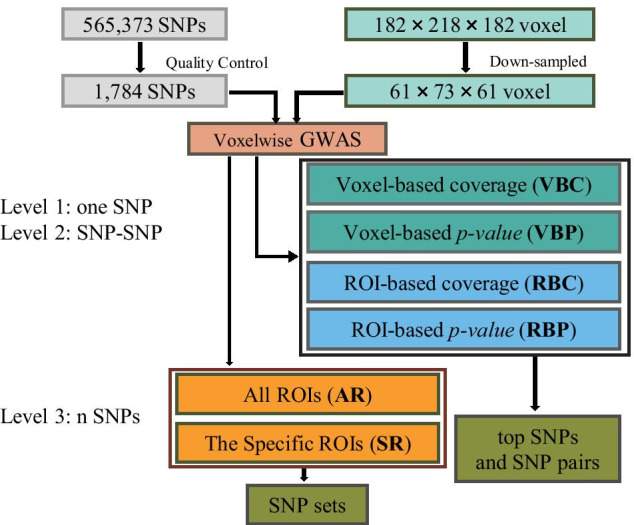
The schematic design of the workflow. VBC = Ranking SNPs according to the number of covered voxels; VBP = Ranking SNPs according to minimally required p threshold; RBC = Ranking SNPs according to the number of ROIs; RBP = Ranking SNPs according to minimally required p threshold; AR = Ranking SNPS according to minimal p threshold covered all ROIS; SR = Ranking SNPS according to minimal p threshold covered a specific ROI; top SNPs, SNP pairs and SNP sets = the identified top SNPs, SNP pairs and SNP sets which have genetic effects with the largest neuroanotomic coverage or specific ROI

Figure 6 shows a schematic design of the workflow of our analyses. In the following subsections, we describe these analyses in more detail.

### Identification and prioritization of single marker effects

**Fig. 7:**
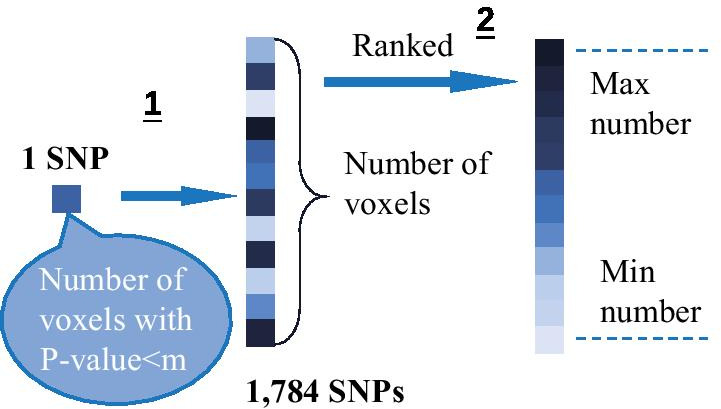
The schematic diagram of ranking SNPs according to the number of covered voxels. 1. Calculate the number of voxels with *p* value less than the set threshold in each SNP; 2. Rank SNPs according to the numbers

VBC: ranking SNPs according to the number of covered voxels. To get the number of voxels, we defined the score for each SNP as the number of voxels who had a *p* value smaller than a threshold. Then we got a list of SNPs sorted by the number of covered voxels in descending order (Fig. 7). To avoid accidental SNPs, we set multiple thresholds. The top 10 frequent SNPs of the results were shown in Fig. 1.

**Fig. 8:**
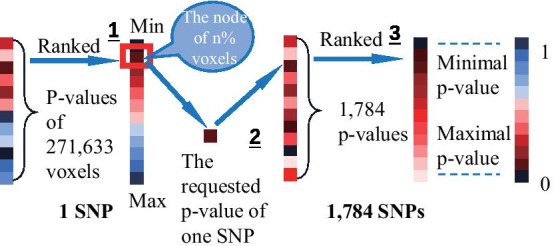
The schematic diagram of ranking SNPs according to minimal *p* value threshold. 1. Rank voxels according to the *p* value; 2. Calculate the *p* value on the set voxel node in each SNP; 3. Rank SNPs according to the *p* values

VBP: ranking SNPs according to minimal *p* value threshold. For finding the minimal *p* value of each SNP in this criterion, we set a condition that the number of voxels was limited (Fig. 8). Figure 1 showed the top 10 SNPs of the results limited by a number of thresholds.

To determine the union of voxels representing the ROI, one way was to calculate the weighted average of the ROI and the other was to select voxels above a certain threshold [39]. In our study, we used the percentile (at least 20%) and *p* value ($p < 0.05$) of voxels as the threshold.

**Fig. 9:**
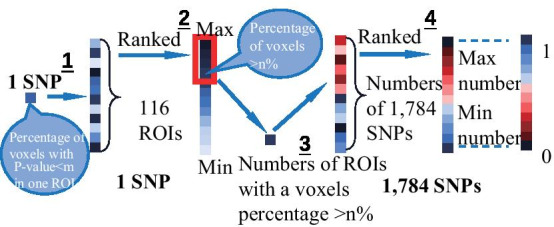
The schematic diagram of ranking SNPs according to number of covered ROIs. 1. Calculate the percentage of voxels with *p* value less than the set threshold in each ROI; 2. Rank ROIs according to the percentages; 3. Calculate the number of ROIs with voxels’ percentage more than the set value in each SNP; 4. Rank SNPs according to the numbers

RBC: ranking SNPs according to number of covered ROIs. Like the VBC, to get the number of covered ROIs, we defined how one SNP affected a ROI. In a similar vein, given a threshold on *p* value, one SNP was considered affecting a ROI if it covered 20% voxels of this ROI. Each SNP was ranked based on the number of the ROIs that it affected (Fig. 9). Figure 1 presented the top 10 SNPs.

**Fig. 10:**
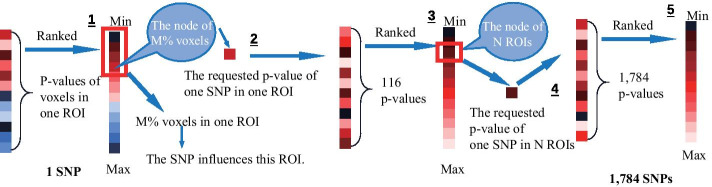
The schematic diagram of ranking SNPs According to minimal *p* value threshold. 1. Rank voxels according to *p* value in each ROI; 2. Calculate the *p* value on the set voxel node; 3. Rank ROIs according to the *p* value; 4. Calculate the *p* value on the set ROI node in each SNP; 5. Rank SNPs according to the *p* value

RBP: ranking SNPs according to minimal *p* value threshold. The goal of this section was to got a minimal *p* value based on ROI. Each SNP was ranked according to the minimal *p* value covered a given number of ROIs. In our experiments, in the condition of covering over 20% voxels of the ROI and the number of the ROI was set, one SNP could be taken into the selection (Fig. 10). The top 10 SNPs with the highest frequent were presented in Fig. 1.

### Identification and prioritization of SNP–SNP effects

For SNP–SNP, a new concept was imported to determine the *p* value within the effects of SNP–SNP. To acquire an operable *p* value of SNP–SNP, we took the minimal *p* value of SNP–SNP on one voxel as the upshot since we used single marker effects. The main strategy of SNP–SNP effects were similar to VBC, VBP, RBC and RBP.

### Identification and prioritization of three SNP effects

**Fig. 11:**
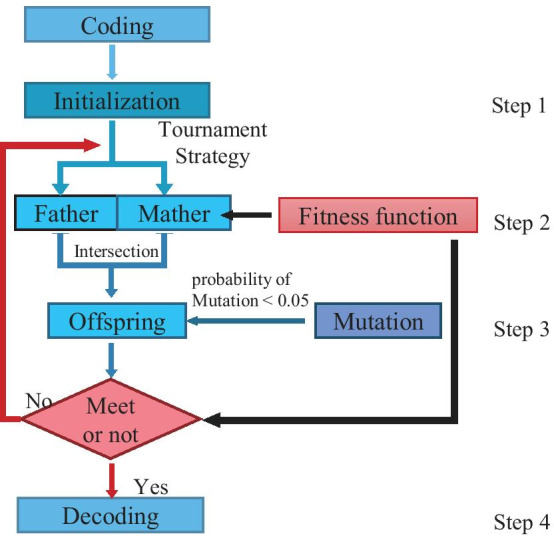
The workflow of the genetic algorithms. Coding = the SNP sets code into a 3-bit code; Initialization = the generation of initial data set; Tournament Strategy = mining the best code from a set of codes; Intersection = exchanging parents’ code in a random site; Mutation = generating a new 3-bit code; Offspring = the result of Intersection or Mutation; Fitness function = based on the *p* value of 3 SNPs affecting 116 ROIs or a specific ROI, the SNPs sets with minimal *p* value are picked out as the parent; Decoding = the 3-bit code decode into corresponding SNP sets

AR: ranking SNPs according to minimal *p* value covered all ROIs. In the effects of the three SNPs, the exhaustive search could take more than 30 days. Therefore, we improved the genetic algorithm to make it more suitable for our experiments. There were four main steps in genetic algorithms (Fig. 11).

Step 1, coding and initialization.

Step 2, fitness function and selection.

Step 3, Intersection and mutation.

Step 4, decoding.

In order to facilitate the analysis, the minimal *p* value of the three SNPs was set on one voxel as the consequence under the same experimental criterion of the two SNPs.

In step 1, considering the set of *p* value on 271,633 voxels of 1784 SNPs, the coding strategy and decoding strategy were established. Since it was the effects of three SNPs, the coding strategy was determined to be a 3-bit code, and each bit was 1 to 1784.

In step 2, to filter out the parent, tournament strategy was introduced in this section. For choosing the parent in the tournament strategy, we defined the fitness function: based on the *p* value of 3 SNPs affecting 116 ROIs, the SNPs sets with larger score were picked out as the parent. The score was defined in formula 1. We first considered the *p* value of SNPs, and then calculated the coverage of SNPs in ROIs. Therefore, when $p value > 0.05$, the proportion of *p* value was 0. When $p = 0.05$, the proportion of coverage and *p* value were same. When $p < 0.05$, the proportion of *p* value was greater than the coverage.

1 $$\begin{aligned} score = {\left\{ \begin{array}{ll} -\log _{10} (p) + \frac{-log_{10} (cov)}{log_{0.05}0.2},&{} p \le 0.05\\ \frac{-log_{10} (cov)}{log_{0.05}0.2},&{} p > 0.05\\ \end{array}\right. } \end{aligned}$$

where *p* is the maximum *pvalue* of 3 SNPs. *cov* is the maximum coverage of 116 ROIs. ${\log _{0.05}0.2}$ is used to modify the score of coverage $= 20\%$ to ${-\log _{10}0.05}$.

In step 3, for yielding progeny populations, a random parameter called the probability of intersection was defined. If it was less than $P_c$, a random position was chosen as the intersection from the 3-bit code for crossover operation. To avoid data locking in a combination, another parameter called probability of variation was generated randomly and compared with $P_m$. If it was less than $P_m$, a mutation operation would be performed.

The adaptive values of Pc and Pm were determined using formula 2 and 3 [40].

2 $$\begin{aligned} P_c= & {} {\left\{ \begin{array}{ll} k_1 \left(f_{max} - f^{'} \right)/\left(f_{max} - {\overline{f}} \right),&{} f^{'} \ge {\overline{f}} \\ k_3,&{} f^{'}<{\overline{f}} \\ \end{array}\right. } \end{aligned}$$

3 $$\begin{aligned} P_m= & {} {\left\{ \begin{array}{ll} k_2 \left(f_{max} - f \right)/\left(f_{max} - {\overline{f}} \right),&{} f \ge {\overline{f}} \\ k_4,&{} f<{\overline{f}} \\ \end{array}\right. } \end{aligned}$$

where $k_1, k_2, k_3, k_4 \le 1.0$. $f_{max}$ is the maximum score of the population. $f^{'}$ is the larger score of two intersecting individuals. ${\overline{f}}$ is the average score of the population. *f* is the score of offspring.

In step 4, the results were decoded into corresponding SNP sets.

SR: ranking SNPs according to minimal *p* value covered a specific ROIs. The fitness function in step 2 was defined as: based on the *p* value of 3 SNPs affecting a sprcific ROI (the coverage > 20% and the biggest coverage of other ROIs < 15%), the SNPs sets with minimal *p* value were picked out as the parent. Other strategy of SR were similar to AR.

## Supplementary Information

**Additional file 1.** The pearson correlation of SNPs, SNP pair or SNP sets and 3168 features.

## Data Availability

Data used in this article was downloaded from the ADNI database (http://adni.loni.usc.edu). Application for access to the ADNI data can be submitted by anyone at http://adni.loni.usc.edu/data-samples/access-data/.

## Abbreviations

AAL: Automatic anatomical labeling
AD: The Alzheimer’s disease
ADNI: The Alzheimer’s disease neuroimaging initiative
AR: All ROIs
EMCI: Early mild cognitive complaint
FGWAS: Functional genome-wide association study
GMD: The gray matter density
GWAS: Voxelwise genome-wide association study
HC: Healthy control
LMCI: Late mild cognitive complaint
MCI: Mild cognitive impairment
MNI: Montreal neurological institute
MRI: Magnetic resonance imaging
PET: Positron emission tomography
PPIX: Protoporphyrinogen IX
RBC: ROI-based coverage
RBP: ROI-based *p* value
ROI: Region-of-interest
SMC: Significant memory concern
SNPs: Single nucleotide polymorphisms
SR: The specific ROIs
VBC: Voxel-based coverage
VBM: Voxel-based morphometry
VBP: Voxel-based *p* value
vGWAS: Voxelwise GWAS
